# Supplementary material for: AI-Driven fetal distress monitoring SDN-IoMT networks
Source: PLoS One. 2025 Jul 31;20(7):e0328099. doi: 10.1371/journal.pone.0328099 (PMC12313076; doi:10.1371/journal.pone.0328099)
Supplement: Attachment — Submitted filename: Comment File.pdf [file pone.0328099.s002.pdf]

# RevisionNotes

This revision notes are disclosed to address comments/reviews on **“PONE-D-24-48368“,** entitled:
**AI-Driven Fetal Distress Monitoring in AI-Native 6G Networks: An SDN-IoMT Framework for Enhanced Medical Connectivity**

**Introduction:**

The authors would like to appreciate the decision that was disclosed by the Editorial Office of the PLOS ONE for providing us an opportunity to revise our manuscript. The suggestions offered by the reviewers have been immensely helpful, and we appreciate your insightful comments on revising the various aspects of the paper. We have revised the manuscript and applied changes as suggested by the reviewers with the best of our performances. In addition, the comments from the reviewers are directly quoted from the decision letter and thereby neither alteration nor rephrasing of the comments was performed.

Finally, the authors attempted as best as possible to strike a balance between conciseness and results reporting. We hope the revised manuscript will better suit the PLOS ONE and we thank you for your continued interest in our research.

## 1stReviewer’s Comments (Highlighted with red font color)

**Comment 1:**

The paper describes the implementation of SDN and DL for fetal data streaming and classification.

**Comment 1a:**

In the current form, it is very unclear the main contribution of the paper.

**Reply to 1st Reviewer’s comment 1a:**

The authors would like to thanks the 1st reviewer for positive feedback. We are truly grateful for the time and effort you invested in reviewing our work and for your thoughtful insights and recommendations. The major objective of this research work is to identify and classify minor class attacks from imbalance data in SDN-IoMT environment using deep learning generative techniques. The main contributions of this paper are as follows:

- To tackle the issue of imbalanced CTG features and clinical data, we have presented a unique GAN-AE-based system in the context of an SDN-IoT environment.To completely examine the proposed model, its performance has been compared with baseline methodologies, including naive DL models and advanced DL models. Every model was evaluated and trained in the same setting to guarantee an equitable comparison.

- The main objective is to improve the monitoring of fetal distress by precisely identifying and categorizing small class abnormalities. To overcome the imbalance in CTG feature data, we utilize the Wasserstein distance-based GAN and reconstruction error inside this framework to

produce believable synthetic data

**Comment 1b:**

It seems to tackle too many complex problems and not one in detail. For example, many information about the 6G network and SDN are provided but no formal tests have been reported.

**Reply to 1st Reviewer's comment 1b:**

The authors have made their efforts to remove the information related to 6G which creates confusion and make this research main contributions unclear.

The manuscript has been revised as follows, with updates made in Introduction Section on pages 1-3.

The medical industry faces challenges in adopting emerging technologies, yet 2 advancements like extended reality (XR), Metaverse, and IoT big data analytics have 3 the potential to revolutionize healthcare [1–3]. These technologies enable advanced services such as telepresence, surgery, and smart diagnostics through IoT-enabled 5 devices [4]. With the integration of artificial intelligence (AI) and deep learning models, 6 networks can process large amounts of medical imaging data in real-time, improving 7 diagnostic accuracy and personalized treatment. These developments are poised to 8 enhance IoT device performance and lead healthcare into a smarter, more connected 9 future [5]………………………………………………………………………………..

The integration of SDN with IoMT, or SDN-IoMT, further improves healthcare 29 infrastructure. By leveraging deep learning, SDN-IoT can analyze vast amounts of data 30 generated by IoT devices in real-time, enabling early disease detection, personalized 31 treatment recommendations, and predictive analytics. Additionally, deep learning can 32 optimize network resource allocation, ensuring efficient data transfer and reduced 33 latency for critical healthcare applications. This approach has the potential to improve patient care quality, streamline healthcare operations, and support proactive, 35 data-driven healthcare solutions [17].

**Comment 1c:**

A Generative model is said to be developed for handling data imbalance, but no results on the quality of the generated data are reported.

**Reply to 1st Reviewer's comment 1c:**

The authors have used the Boundary Equilibrium GAN (BEGAN) model for synthetic data generation. BEGAN model works on the principal of reconstruction error which maintains the balance between generator and descrimintor. This reconstruction error property guarantees the quality of data.

The manuscript has been revised as follows on pages 15 and 17.

For binary classification the proposed system produced 6,000 synthetic data points for each class by using a trained GAN. For multiclassification, the proposed approach produces AI data for minor classes that include less than 10% of the total population. Consequently, a trained generative model generates 10,000 synthetic data points for both the suspicious and pathological classifications.

**Comment 2:**

Finally, DL models are compared to determine fetal distress but no clear information about the gold standard are reported.

**Reply to 1st Reviewer's comment 2:**

In this study, the authors used three types of model for rigrious testing and comprehensive analysis of the proposed technique. Baseline deep learning models (CNN, DNN, LSTM), advanced deep learning models (CNN_ae) and proposed technique (G.CNN_ae). The results show that the proposed model achieved highest accuracy and f1-score against baseline and advanced deep learning models in case of multiclassification. Results are shown in a table 6.

| Classifier | A | Suspicious | | | Pathological | | |
|---|---|---|---|---|---|---|---|
| | | R | P | F1 | R | P | F1 |
| $DNN$ | 80.7 % | 6.8% | 78.5% | 10.3% | 5.6% | 76.9% | 9.8% |
| $CNN$ | 81.2 % | 8.3% | 83.2% | 13.4% | 7.3% | 81.8% | 12.5% |
| $LSTM$ | 83.7 % | 8.1% | 80.4% | 12.3% | 6.3% | 78.9% | 11.9% |
| $DNN_{AE}$ | 89.4 % | 9.3% | 87.5% | 17.5% | 8.9% | 85.5% | 15.2% |
| $CNN_{AE}$ | 89.6 % | 12.3% | 88.3% | 19.3% | 10.4% | 87.7% | 17.8% |
| $G.LSTM$ | 88.8 % | 12.9% | 85.9% | 21.6% | 11.3% | 83.8% | 19.1% |
| $G.DNN_{AE}$ | 93.3 % | 14.3% | 84.3% | 24.1% | 13.5% | 82.0% | 22.5% |
| $G.CNN_{AE}$ | 94.2 % | 13.9% | 85.8% | 23.6% | 12.6% | 82.0% | 21.1% |

**A** = Accuracy, **R** = Recall, **P** = Precision, **F1** = F1-score, **G.LSTM**, **G.DNN**$_{AE}$, **G.CNN**$_{AE}$ = Proposed Framework

**Table 6.** Performance comparison of classifiers for Suspicious and Pathological categories.

**Comment 3:**

performance metrics not clear.

**Reply to 1st Reviewer's comment 3:**

The authors have used four performance metrices: Accuracy, Precision, Recall, and F1-score.

The manuscript has been revised as follows, with updates made in Experimentation and Analysis

Section on pages 15.

- Accuracy:

$$Accuracy = \frac{TP + TN}{TP + FP + TN + FN}$$

- Precision:

$$Precision = \frac{TP}{TP + FP}$$

- Recall:

$$Recall = \frac{TP}{TP + FN}$$

- F1-score

$$F1 - score = 2 * \frac{Precision * Recall}{Precision + Recall}$$

**Comment 4:**

model selection performed on the test set, which is a well-known methodological mistake. In addition, the paper requires extensive revision for clarity.

**Reply to 1st Reviewer's comment 4:**

The authors have split the dataset into 7:3 ratio for training and testing the proposed model. Following that, an 8:2 ratio is used to further split the training data into training and validation data. For trainig the proposed model, real data is combined with the synthic data for extensive model training. Once the model is fully trained, it is tested on real 30% test dataset for unbiased results. The authors have made their best efforts to do the extensive revisions for the clarity purpose.

The manuscript has been revised as follows, with updates made in Experimentation and Analysis Section on page 13.

A 7:3 ratio is used to divide the dataset into training and test data. Following that, an 8:2 ratio is used to further split the training data into training and validation data.

**Comment 5:**

Not clear who and how were defined the categories for fetal distress.

**Reply to 1st Reviewer's comment 5:**

The authors defined the fetal distress categories into three types: Normal, suspicious, and pathological. The fetal distress categories are taken from the existing literature. The reference is mentioned below in which fetal distress categories are described in section 1.4.32.

**References to 1st Reviewer's comment 5:**

https://www.nice.org.uk/guidance/ng229/chapter/Recommendations.

**Comment 5:**

The performance metrics reported are suitable for binary classification. However, the problem tackled was a 3-class classification problem. Not sure authors computed exactly these quantitities. At a certain point, the problem becomes a binary one. Not clear at all.

**Reply to 1st Reviewer's comment 5:**

For comprehensive training and testing the model, the authors have performed two types of classification: Binary classification and multi-classification. In binary classification, the data is divided into two classes Normal and Abnormal data. Where as in multi-classification, the data is divided into three classes. Table 6 gives the result of multi-classifiction.

| Classifier | A | Suspicious | | | Pathological | | |
|---|---|---|---|---|---|---|---|
| | | **R** | **P** | **F1** | **R** | **P** | **F1** |
| $DNN$ | 80.7 % | 6.8% | 78.5% | 10.3% | 5.6% | 76.9% | 9.8% |
| $CNN$ | 81.2 % | 8.3% | 83.2% | 13.4% | 7.3% | 81.8% | 12.5% |
| $LSTM$ | 83.7 % | 8.1% | 80.4% | 12.3% | 6.3% | 78.9% | 11.9% |
| $DNN_{AE}$ | 89.4 % | 9.3% | 87.5% | 17.5% | 8.9% | 85.5% | 15.2% |
| $CNN_{AE}$ | 89.6 % | 12.3% | 88.3% | 19.3% | 10.4% | 87.7% | 17.8% |
| $G.LSTM$ | 88.8 % | 12.9% | 85.9% | 21.6% | 11.3% | 83.8% | 19.1% |
| $G.DNN_{AE}$ | 93.3 % | 14.3% | 84.3% | 24.1% | 13.5% | 82.0% | 22.5% |
| $G.CNN_{AE}$ | 94.2 % | 13.9% | 85.8% | 23.6% | 12.6% | 82.0% | 21.1% |

**A** = Accuracy, **R** = Recall, **P** = Precision, **F1** = F1-score, **G.LSTM**, **G.DNN**$_{AE}$, **G.CNN**$_{AE}$ = Proposed Framework

**Table 6.** Performance comparison of classifiers for Suspicious and Pathological categories.

**Comment 6:**

What is the objective of the authors with "we published the results based on the model that achieved the best detection rate in the test dataset"? Doing so, the test set becomes another validation set because selection is performed on these data. It is a methodological mistake if not well motivated.

**Reply to 1st Reviewer's comment 6:**

The authors have addressed the comment 3 which is similar to this comment 6 and provided the answer which is given below.

The manuscript has been revised as follows, with updates made in Experimentation and Analysis Section on page 13.

A 7:3 ratio was used to divide the 360 dataset into training and test data. Following that, an 8:2 ratio was used to further split 361 the training data into training and validation data.

**Comment 7:**

Figure 1 is never mentioned in the text.

**Reply to 1st Reviewer's comment 7:**

The authors apologize for such type of mistakes. The authors have mentioned the Figure 1 in the text.

The manuscript has been revised as follows, with updates made in Introduction Section on page 3.

The application of DL models for anomaly detection and classification in SDN-IoT medical contexts is shown in Fig. 1.

**Comment 8:**

Figure 2 shows many CTG devices connected and able to stream their data. However, I am not aware of any commercial device which can do that.

**Reply to 1st Reviewer's comment 8:**

The authors agree with the 1st reviewer that there is no such type of commercial CTG device that is able to stream its data online. The authors have taken the results from traditional CTG machines avaialables in hospitals. Figure 2 is basically a symbolic representation of CTG devices like others IoMT devices.

**Comment 9:**

Please check the instruction for authors since the table of acronyms is rarely allowed by journals. Also, check all acronyms are instantiated once and in the right order (e.g. DL is used before instantiation)

**Reply to 1st Reviewer's comment 9:**

The authors agree with the 1st reviewer, I have carefully reviewed the entire manuscript to ensure that all acronyms are introduced in the correct order, with each acronym being instantiated before its first occurrence in the text.

**Comment 10:**

What is BV?

**Reply to 1st Reviewer's comment 10:**

BV stands for "Baseline Variability" which is one of the CTG most important feature.

**Comment 11:**

Are authors sure that the GAN proposed in [21] is called "Reconstruction Error"?

**Reply to 1st Reviewer's comment 11:**

Yes, GAN proposed in [21] is a TS-GAN which can work on reconstruction error, but it depends on the specific use case and implementation. For example reconstruction error is often used to identify anomalies in time-series data. If the reconstructed time series from the generator or encoder-decoder network significantly deviates from the original, it can indicate an anomaly.

**Comment 12:**
What is FHR's decentralization?

**Reply to 1st Reviewer's comment 12:**
The FHR (Fetal Heart Rate) decentralization typically refers to a decentralized pattern observed in the Fetal Heart Rate during CTG monitoring, often indicating potential issues with fetal well-being. In the context of medical interpretation, FHR decentralization describes certain repetitive changes in the fetal heart rate that deviate from the baseline and are associated with contractions or other factors.

## 2ndReviewer's Comments (Highlighted with blue font color)

**Comment 1:**
The Sensing layer described is only meant to detect fetal cardiac activity and some maternal vital signs. It does not detect uterine contractions. While fetal cardiac activity is interpreted based on uterine contraction. The sensing should be able or developed to detect uterine contractions as well.

**Reply to 2nd Reviewer's comment:**
The authors would like to thanks the 2nd reviewer for positive feedback. We are truly grateful for the time and effort you invested in reviewing our work and for your thoughtful insights and recommendations. Yes, authors agree with the 2nd reviewer that fetal cardiac activity is interpreted based on uterine contraction so that authors have added and performed the sensing of the uterine contractions to make the model more efficient and robust.

According to the 2nd reviewer suggestions,

The manuscript has been updated and revised as follows:

FHR, deceleration, and uterine contractions are the three key characteristics of CTG that are predominantly discussed in this article, along with other medical measures like blood pressure, temperature, and oxygen levels that are related to the health of mothers. These metrics are crucial for keeping an eye on fetal distress. The most crucial measures are FHR, deceleration, and uterine contractions since any unsettling aspect of either calls for quick action from medical professionals.

**Comment 2:**

Why did was median used against mean to replace the missing data dst and bst?

**Reply to 2nd Reviewer's comment 2:**

The choice of median over mean for replacing missing data is made because the median is more robust to outliers and skewed distributions.

**Comment 3:**

Blood pressure (BP) of 139/99 is not normal (Table 2). Any diastolic BP greater that 89mmHg is High. With this AI devise women with hypertensive disorders of pregnancy may be missed in the antenatal or labour period.

**Reply to 2nd Reviewer's comment 3:**

The authors thank the 2nd reviewer for pointing out BP. We acknowledge that a BP of 139/99 mmHg is indeed classified as hypertensive, as diastolic BP greater than 89 mmHg falls into the category of high BP. We have corrected the value in Table 1 to reflect this classification. Additionally, we have updated the model so that AI device includes specific thresholds and parameters to detect hypertensive disorders in pregnancy to ensure such cases are not missed during the antenatal or labor period.

According to the 2nd reviewer suggestions,

The manuscript has been updated and revised as follows:

**Table 1.** A description of CTG and clinical features, including the types of sensors used to capture these features and the normal range for each feature.

| Sr. No. | Feature Name | Sensor Type | Normal Range |
|---|---|---|---|
| 1 | Maternal Blood Pressure | B.P Sensor | $\leq 139/89$ |
| 2 | Maternal Heart Rate | Heart-beat Sensor | $< 90/\text{min}$ |
| 3 | Maternal Oxygen Saturation (%) | Oxygen Level Sensor | $\geq 90\%$ |
| 4 | Maternal Temperature | Temp. Sensor | 97–99 F |
| 5 | Fetal Heart Rate | Fetal Heart Rate Sensor | 110–160 |
| 6 | Baseline Variability | Baseline Variability Sensor | 5–25 |
| 7 | Acceleration | Acceleration Sensor | 2 Acc. per 20 minutes |
| 8 | Deceleration | Deceleration Sensor | 0 |

**Comment 4:**

For more information on Blood pressure in pregnancy, check guidelines by International Society for the Study of Hypertension in Pregnancy and FIGO.

**Reply to 2nd Reviewer's comment 4:**

The authors have taken this comment on board and thanks the 2nd reviewer for providing a useful link.

**Comment 5:**

Other comments and corrections are in the text.

**Comment 5a:**

In correct spelling of "pediatrics"

**Reply to 2nd Reviewer's comment 5a:**

According to the reviewer suggestions;

The manuscript has been updated and revised as follows:

Gynecologists and pediatricians then review the patient's classification to decide on the next best step.

**Comment 5b:**

In correct spelling of "Experimentation ans Analysis"

**Reply to 2nd Reviewer's comment 5b:**

According to the reviewer suggestions;

The manuscript has been updated and revised as follows:

Experimentation and Analysis

**Comment 5c:**

In correct spelling of "139/99"

**Reply to 2nd Reviewer's comment 5c:**

According to the reviewer suggestions;

The manuscript has been updated and revised as follows:

**Table 1.** A description of CTG and clinical features, including the types of sensors used to capture these features and the normal range for each feature.

| Sr. No. | Feature Name | Sensor Type | Normal Range |
|---|---|---|---|
| 1 | Maternal Blood Pressure | B.P Sensor | $\leq 139/89$ |
| 2 | Maternal Heart Rate | Heart-beat Sensor | $< 90$/min |
| 3 | Maternal Oxygen Saturation (%) | Oxygen Level Sensor | $\geq 90\%$ |
| 4 | Maternal Temperature | Temp. Sensor | 97–99 F |
| 5 | Fetal Heart Rate | Fetal Heart Rate Sensor | 110–160 |
| 6 | Baseline Variability | Baseline Variability Sensor | 5–25 |
| 7 | Acceleration | Acceleration Sensor | 2 Acc. per 20 minutes |
| 8 | Deceleration | Deceleration Sensor | 0 |
