# Supplementary material for: AI-Driven fetal distress monitoring SDN-IoMT networks
Source: PLoS One. 2025 Jul 31;20(7):e0328099. doi: 10.1371/journal.pone.0328099 (PMC12313076; doi:10.1371/journal.pone.0328099)
Supplement: Attachment — Submitted filename: review.pdf [file pone.0328099.s003.pdf]

Title of the Manuscript:

AI-Driven Fetal Distress Monitoring SDN-IoMT Networks
Manuscript Number: PONE-D-24-48368R1

General Assessment:

This manuscript presents a technically innovative approach combining Software-Defined Networking (SDN), Internet of Medical Things (IoMT), and Deep Learning (DL) for fetal distress monitoring using cardiotocography (CTG) data. The authors propose a GAN-Autoencoder framework designed to mitigate the common issue of class imbalance in CTG datasets. The work is well-structured and technically sound, with appropriate performance metrics and comparisons to state-of-the-art models. However, several methodological and clinical aspects require clarification and expansion, particularly regarding clinical integration, use of standardized CTG scoring systems, and the variability of real-world signals.

Strengths:

- Addresses a clinically relevant problem — early detection of fetal distress.
- Applies an original combination of GANs and Autoencoders to synthetic data generation for minority CTG classes.
- Strong comparative analysis against both naïve and advanced deep learning models.
- Use of public CTG datasets (CTU-UHB) enhances transparency and reproducibility.

Major Comments:

1. **Clinical Relevance & Real-World Use:**
   The manuscript would benefit from a clearer discussion of how the proposed framework could be integrated into clinical workflows. Is the system designed to function in real-time at the bedside? Have obstetricians or maternal-fetal medicine specialists been consulted or involved in model validation?

2. **Interpretability & Feature Importance:**
   While the model performs well in overall metrics, it is not clear which CTG or clinical features contributed most significantly to classification. Feature importance or SHAP analysis would strengthen clinical interpretability.

3. **Evaluation Strategy:**
   Please clarify whether cross-validation (e.g., 10-fold) was used, particularly for evaluation of minority classes (e.g., pathological). With high data imbalance, this is crucial to avoid overfitting or optimistic metrics.

4. **Reproducibility:**
   The dataset is shared via Figshare, which is commendable. However, the source code for model implementation is not provided. PLOS ONE encourages full reproducibility. A link to a GitHub repository or similar would be highly beneficial.

5. **Minor Class F1-Scores:**
   Although the overall accuracy is high, F1-scores for the pathological class remain low (~21.1%). This should be acknowledged more explicitly in the limitations section.

6. **Training Data Size for Minority Classes:**
   While the total training set includes 29,400 samples, only 500 are pathological and 970 are suspicious. This small number of true examples in minority classes is a limitation, even if augmented by GAN-generated data. The authors should discuss the potential for overfitting and limitations in generalizability due to the low number of real-world samples in these critical categories.

7. **Real-World Variability in CTG Signals:**
   As CTG signals may vary in response to real-time clinical decisions and external maternal factors (e.g., position changes or stimulation protocols), future validation of the model in actual clinical environments would be valuable to assess its robustness under such dynamic conditions.

8. **Applicability to Intrapartum vs Antepartum Monitoring:**
   Based on the manuscript, it appears that the proposed framework is focused on antepartum monitoring. However, CTG patterns and fetal response may differ significantly during labor due to uterine activity and stress. The authors should clarify whether the model is intended solely for prenatal (antepartum) use or if it has been tested or could be adapted for intrapartum monitoring scenarios.

9. **Use of Standardized CTG Scoring Systems:**
   The study does not refer to any internationally accepted classification system for CTG interpretation, such as the Fisher score (including Krebs modification) or FIGO guidelines. Instead, it relies on author-defined thresholds for FHR, variability, accelerations, and decelerations. This may hinder clinical applicability and comparability with real-world obstetric practice. The authors should clarify whether their classification logic was based on, inspired by, or mapped to any established guidelines. If not, justification and discussion of the implications of using a custom classification system should be included.

10. **Lack of Reference to Dawes-Redman Criteria or STV/LTV Parameters:**
   The manuscript does not reference the Dawes-Redman criteria, a widely used and validated computerized system for antepartum CTG analysis. While the dataset used may include features such as short-term variability (STV), these were not described among the model's input features. STV and long-term variability (LTV) are core components of Dawes-Redman interpretation. The authors should clarify whether such features were excluded, and why. Including STV/LTV or aligning their classification logic with recognized systems could strengthen the clinical utility and

comparability of the model.

11. **Exclusion of Sinusoidal Pattern as a CTG Abnormality:**
A sinusoidal fetal heart rate pattern is widely recognized in clinical obstetrics as a highly pathological CTG feature, often associated with severe fetal compromise (e.g., fetal anemia or hypoxia). However, the manuscript makes no reference to sinusoidal patterns, nor is there any indication that such a feature was included in the dataset or evaluated during classification. The authors should clarify whether this pattern was observed or represented in the dataset, and if not, acknowledge this as a limitation regarding model generalizability to rare but critical CTG abnormalities.

Minor Comments:

- Temperature Units: In Table 1, maternal body temperature is listed only in Fahrenheit (97–99°F). Please provide the Celsius equivalent (approximately 36.1–37.2 °C) for clarity, especially for international readers from regions using the metric system.

- There are several minor English language errors in the abstract and introduction (e.g., "due to complexity and heterogeneous" → should be "due to complexity and heterogeneity"). A careful grammar and style check is recommended.

- Specify which version of the OpenFlow protocol is assumed in the SDN communication layer.

- Figures 2 and 3 could benefit from clearer axis labeling and a more consistent visual style.

Recommendation to the Editor:

Major Revision

The manuscript presents a promising AI framework with strong technical merit. However, to meet PLOS ONE's standards for clarity, reproducibility, and applicability, the authors should address the clinical context more explicitly, enhance transparency of implementation, and revise for clarity and international accessibility. The limited size of minority class training data, variability of CTG signals, absence of standardized interpretation criteria, and unclear use of STV/LTV features and sinusoidal patterns require further clarification.
