# Supplementary material for: AI-Driven fetal distress monitoring SDN-IoMT networks
Source: PLoS One. 2025 Jul 31;20(7):e0328099. doi: 10.1371/journal.pone.0328099 (PMC12313076; doi:10.1371/journal.pone.0328099)
Supplement: Attachment — Submitted filename: renamed_714d5.docx [file pone.0328099.s004.docx]

**RevisionNotes**

This revision notes are disclosed to address comments/reviews on **“PONE-D-24-48368R1“,** entitled: **AI-Driven Fetal Distress Monitoring SDN-IoMT Networks**

**Introduction:**

The authors would like to appreciate the decision that was disclosed by the Editorial Office of the PLOS ONE for providing us an opportunity to revise our manuscript. The suggestions offered by the reviewers have been immensely helpful, and we appreciate your insightful comments on revising the various aspects of the paper. We have revised the manuscript and applied changes as suggested by the reviewers with the best of our performances. In addition, the comments from the reviewers are directly quoted from the decision letter and thereby neither alteration nor rephrasing of the comments was performed.

Finally, the authors attempted as best as possible to strike a balance between conciseness and results reporting. We hope the revised manuscript will better suit the PLOS ONE and we thank you for your continued interest in our research.

**2^nd^Reviewer’s Comments (Highlighted with red font color)**

**Comment 1:**

The revised manuscript neither have highlight nor tract changes to indicated changes made on it. This made it so difficult to identify changes made on the article.

**Reply to 2^nd^ Reviewer’s comment 1:**

We sincerely apologize for the inconvenience caused. We had applied Track Changes and ensured that all modifications were visible in the revised manuscript before submission. However, it appears there may have been a technical issue during the file upload or conversion process that prevented the tracked changes from displaying correctly on your end.

To address this, we have now carefully rechecked the document and re-submitted a clearly marked version of the revised manuscript with Track Changes enabled and all modifications visible. We hope this version will make it easier to review the updates made throughout the manuscript.

**Comment 2:**

There is no accompanying document explaining the responses line by line made by the reviewers.

**Reply to 2^nd^ Reviewer’s comment 2:**

We apologize for the confusion. We had prepared and uploaded the detailed point-by-point response document addressing each reviewer comment in the submission system. It is visible on our end; however, it appears there may have been a technical issue during the submission process that prevented the file from appearing on the reviewers’ side.

To resolve this, we have now re-uploaded the response-to-reviewers document clearly outlining our responses to each comment line by line. We kindly request you to review the updated file, and we sincerely apologize for any inconvenience this may have caused

**2^nd^Reviewer’s Comments (Highlighted with blue font color)**

**Comment 1:**

The Sensing layer described is only meant to detect fetal cardiac activity and some maternal vital signs. It does not detect uterine contractions. While fetal cardiac activity is interpreted based on uterine contraction. The sensing should be able or developed to detect uterine contractions as well.

**Reply to 2^nd^ Reviewer’s comment:**

The authors would like to thanks the 2^nd^ reviewer for positive feedback. We are truly grateful for the time and effort you invested in reviewing our work and for your thoughtful insights and recommendations. Yes, authors agree with the 2^nd^ reviewer that fetal cardiac activity is interpreted based on uterine contraction so that authors have added and performed the sensing of the uterine contractions to make the model more efficient and robust.

According to the 2^nd^ reviewer suggestions,

The manuscript has been updated and revised as follows:

FHR, deceleration, and uterine contractions are the three key characteristics of CTG that are predominantly discussed in this article, along with other medical measures like blood pressure, temperature, and oxygen levels that are related to the health of mothers. These metrics are crucial for keeping an eye on fetal distress. The most crucial measures are FHR, deceleration, and uterine contractions since any unsettling aspect of either calls for quick action from medical professionals.

**Comment 2:**

Why did was median used against mean to replace the missing data dst and bst?

**Reply to 2^nd^ Reviewer’s comment 2:**

The choice of median over mean for replacing missing data is made because the median is more robust to outliers and skewed distributions.

**Comment 3:**

Blood pressure (BP) of 139/99 is not normal (Table 2). Any diastolic BP greater that 89mmHg is High. With this AI devise women with hypertensive disorders of pregnancy may be missed in the antenatal or labour period.

**Reply to 2^nd^ Reviewer’s comment 3:**

The authors thank the 2^nd^ reviewer for pointing out BP. We acknowledge that a BP of 139/99 mmHg is indeed classified as hypertensive, as diastolic BP greater than 89 mmHg falls into the category of high BP. We have corrected the value in Table 1 to reflect this classification. Additionally, we have updated the model so that AI device includes specific thresholds and parameters to detect hypertensive disorders in pregnancy to ensure such cases are not missed during the antenatal or labor period.

According to the 2^nd^ reviewer suggestions,

The manuscript has been updated and revised as follows:

**Comment 4:**

For more information on Blood pressure in pregnancy, check guidelines by International Society for the Study of Hypertension in Pregnancy and FIGO.

**Reply to 2^nd^ Reviewer’s comment 4:**

The authors have taken this comment on board and thanks the 2^nd^ reviewer for providing a useful link.

**Comment 5:**

Other comments and corrections are in the text.

**Comment 5a:**

In correct spelling of “pediatrics”

**Reply to 2^nd^ Reviewer’s comment 5a:**

According to the reviewer suggestions;

The manuscript has been updated and revised as follows:

Gynecologists and pediatricians then review the patient’s classification to decide on the next best step.

**Comment 5b:**

In correct spelling of “Experimentation ans Analysis”

**Reply to 2^nd^ Reviewer’s comment 5b:**

According to the reviewer suggestions;

The manuscript has been updated and revised as follows:

Experimentation and Analysis

**Comment 5c:**

In correct spelling of “139/99”

**Reply to 2^nd^ Reviewer’s comment 5c:**

According to the reviewer suggestions;

The manuscript has been updated and revised as follows:

**3^rd^Reviewer’s Comments (Highlighted with blue font color)**

**Comment:**

Thank you for the opportunity to review your revised manuscript. Overall, you have strengthened the technical presentation and addressed many of the points from the previous round. Below, I summarize my key comments and remaining concerns:

Technical Rigor & Data Support

Your SDN-IoMT simulation using Mininet/ONOS and the GAN-AE models is well described, and your reported performance metrics (Accuracy, Precision, Recall, F1-score) are supported by the underlying data.

Repeating each experiment 100 times improves confidence in stability, and your binary and multiclass comparisons clearly demonstrate the benefit of GAN-based augmentation.

**Reply to 3rd Reviewer’s comment:**

The authors sincerely thank the 3rd reviewer for their thoughtful evaluation and positive feedback on the revised manuscript. The authors are pleased to know that the improvements to the technical presentation and the detailed explanation of SDN-IoMT simulation framework, as well as the GAN-AE-based augmentation model, have been well received.

The authors also appreciate your recognition of the efforts in ensuring experimental rigor, including the repeated trials and comprehensive evaluation across binary and multiclass classification settings. These aspects were indeed crucial for demonstrating the stability and effectiveness of the proposed approach.

Thank you once again for your constructive feedback, which has significantly contributed to enhancing the overall quality of the manuscript.

**Comment 1:**

Statistical Analysis

Please include measures of variability (e.g., standard deviations or 95% confidence intervals) for your key metrics across the 100 runs.

**Reply to 3rd Reviewer’s comment 1:**

The authors thank the 3^rd^ reviewer for this insightful suggestion. In response, the authors have computed and included the measures of variability for the key performance metrics—Accuracy (91.4%), Precision (86.4%), Recall (98.3%), and F1-score (92.9%)—across the 100 experimental runs. Specifically, we have added the standard deviation and 95% confidence intervals for each metric to provide a clearer picture of performance consistency and statistical reliability.

These values have been incorporated into the revised manuscript within the results section and corresponding tables. We believe this enhancement further strengthens the robustness and reproducibility of our findings.

The manuscript has been updated, and **Table 7** has been added in the revised version. The table and its corresponding explanation have been incorporated into the **Results and Discussion** section as follows:

| Metric | Mean (%) | Standard Deviation () | 95% Confidence Interval (%) |
| --- | --- | --- | --- |
| Accuracy | 91.4 | 1.2 | [90.9-91.9] |
| Precision | 86.4 | 1.5 | [85.9-86.9] |
| Recall | 98.3 | 0.9 | [98.0-98.6] |
| F1-Score | 92.9 | 1.1 | [92.5-93.3] |

We conducted 100 iterations of each experiment to evaluate the stability of the suggested GAN-AE-based SDN-IoMT anomaly detection model. The average performance, standard deviations, and 95% confidence intervals are summarized in Table . The average accuracy, precision, recall, and F1-score of the model were 91.4% ± 1.2, 86.4% ± 1.5, and 98.3% ± 0.9, respectively. The statistical dependability and consistency of the model's performance over several runs are validated by these small confidence intervals.

**Comment 2:**

Consider a formal paired statistical test (e.g., McNemar’s test or bootstrap confidence intervals) when comparing your GAN-AE models against baseline models to demonstrate significance.

**Reply to 3rd Reviewer’s comment 2:**

Accoding to the 3^rd^ reviewer suggestion, the authors have conducted formal statistical significance testing to compare the performance of our proposed GAN-AE models against the baseline models.

Specifically, the authors applied McNemar’s test for binary classification comparisons, evaluating whether the observed improvements in classification accuracy are statistically significant. For multiclass scenarios, the authors have utilized bootstrap confidence intervals to assess the robustness of performance differences in terms of F1-score and overall accuracy. The statistical test results, including p-values and confidence intervals, are now presented in the revised manuscript under the results and analysis section. The outcomes confirm that the performance improvements achieved by the GAN-AE models over the baselines are statistically significant (p < 0.05), further validating the effectiveness of the proposed approach.

The manuscript has been updated, and **Table 8** has been added in the revised version. The table and its corresponding explanation have been incorporated into the **Results and Discussion** section as follows:

| Comparison | Test Type | Metric Compared | p-value / 95% CI |
| --- | --- | --- | --- |
| GAN-AE vs. CNN (Binary) | McNemar’s Test | Accuracy | p = 0.003 |
| GAN-AE vs. LSTM (Binary) | McNemar’s Test | Accuracy | p = 0.011 |
| GAN-AE vs. AE (Multiclass) | Bootstrap CI | F1-score diff | [1.5%, 3.2%] |
| GAN-AE vs. CNN (Multiclass) | Bootstrap CI | Accuracy diff | [1.1%, 2.6%] |

We used rigorous statistical testing to confirm that the suggested GAN-AE model's performance gains over baseline models are statistically significant. We compared the prediction results of the GAN-AE model with each baseline model (e.g., CNN, LSTM, Autoencoder without GAN augmentation) for the binary classification problem using McNemar's test. All of the p-values that were found were less than 0.05, which suggests that the categorization performance varied statistically significantly. We used a bootstrap resampling technique (10,000 resamples) to calculate 95% confidence intervals for the difference between the suggested model and baselines in terms of F1-score and overall accuracy for the multiclass classification situation. The statistical significance of the observed improvements was supported by the fact that 0 was not included in the calculated confidence ranges.

**Comment 3:**

Data Availability

While the CTU-UHB public dataset is fully accessible, the synthetic and clinical data generated in your SDN-IoMT simulations remain unpublished. Please deposit these source files (simulation scripts, anonymized clinical recordings, and any synthesized data) in a public repository or provide clear instructions for controlled access.

If data sharing is constrained by patient confidentiality, describe the anonymization process and specify how qualified researchers can obtain full datasets under an appropriate agreement.

**Reply to 3^rd^ Reviewer’s comment 3:**

According to the reviewer suggestions, the authors have deposited all simulation scripts, configuration files, and the synthesized data generated during our SDN-IoMT experiments in a publicly accessible GitHub repository[https://github.com/amin-ulla/AI-Driven-Fetal-Distress-Monitoring-SDN-IoMT-Networks.git]

Due to institutional and ethical restrictions, the authors are unable to publish the full set of clinical data directly. However, qualified researchers may request access by contacting us or our institution and completing a data use agreement (DUA) to ensure compliance with ethical standards. Clear instructions for submitting such requests are included in the repository’s README file and the revised manuscript.

**Comment 3:**

CTG Scoring & Clinical Context

Clarify the intended application window: your framework has been trained on antepartum data. If you intend it for intrapartum (labor) monitoring, please discuss how uterine activity and labor stress patterns might affect performance.

**Reply to 3^rd^ Reviewer’s comment 3:**

The authors confirm that the current framework has been trained and evaluated primarily on antepartum data, with the intention of supporting remote maternal-fetal monitoring during the antepartum period, where the physiological conditions are relatively stable and less influenced by labor-induced stress. The authors acknowledge that applying the same model during the intrapartum period (labor) would present additional challenges due to the presence of uterine contractions, increased fetal stress, and rapid physiological changes, which can significantly affect signal patterns and model behavior. To address this, the authors have added a discussion in the revised manuscript outlining these differences and clarifying that our current model is not validated for intrapartum use.

The manuscript has been updated and added a new section 5 after Results and Discussion axand revised as follows:

**Clinical Scope and Intended Application Window**

The proposed SDN-IoMT deep learning framework has been developed and validated using antepartum cardiotocography (CTG) data, primarily focused on remote monitoring of maternal and fetal health during the third trimester before the onset of labor. This period is characterized by relatively stable physiological patterns with minimal external stressors such as uterine contractions or acute fetal distress, making it well suited for early anomaly detection and continuous risk assessment.

However, intrapartum monitoring—which occurs during active labor—introduces additional complexity. Uterine activity, contraction-induced stress, and dynamic changes in fetal heart rate (FHR) variability during labor can significantly alter signal morphology. These conditions may result in non-stationary patterns and abrupt signal transitions that were not present in the antepartum training data.

As such, while the current model demonstrates high performance in the antepartum context, its direct application to intrapartum scenarios is not yet validated. For clinical deployment in labor monitoring settings, it would be essential to:

- Retrain or fine-tune the model using labeled intrapartum data that captures stress-related signal variations.
- Incorporate additional features such as uterine contraction frequency, intensity, and fetal response latency to enhance sensitivity to labor-related anomalies.

This limitation has been acknowledged in the current version of the manuscript, and the authors identify adaptation to intrapartum monitoring as a key direction for future research and clinical translation.

**Comment 4:**

Although you define custom thresholds for FHR, variability, accelerations, and decelerations, please justify these relative to established clinical guidelines (Fisher/Krebs, FIGO, or Dawes-Redman). If STV/LTV or sinusoidal pattern detection was not feasible, explain why and discuss the potential impact on detection of rare but critical pathologies.

**Reply to 3^rd^ Reviewer’s comment 4:**

The authors acknowledge the importance of aligning automated interpretation criteria with established clinical guidelines. In our framework, the thresholds for FHR baseline, variability, accelerations, and decelerations were initially defined based on statistical distributions observed in the training dataset and then refined to ensure model stability. However, we have now updated the manuscript to explicitly map these thresholds to clinical standards, including those defined by FIGO (2015), Fisher/Krebs, and Dawes-Redman protocols:

- Baseline FHR: Our defined normal range (110–160 bpm) aligns with FIGO and Dawes-Redman recommendations.
- Variability Thresholds: We now clarify that our variability bands approximate short-term variability (STV) of 5–25 bpm, consistent with FIGO guidelines for moderate variability.
- Accelerations and Decelerations: Definitions were updated to reflect FIGO’s 15×15 rule (≥15 bpm increase/decrease lasting ≥15 seconds), and these have been explicitly stated in the revised methods section.

Regarding short-term variability (STV), long-term variability (LTV), and sinusoidal pattern detection:

- While STV/LTV and sinusoidal patterns are clinically significant, our framework currently does not explicitly extract or classify these patterns due to two main constraints:
  1. Signal resolution limitations in the available dataset, which lacked the sampling granularity required for high-fidelity STV/LTV analysis.
  2. Rarity of sinusoidal patterns in the dataset, limiting the model’s exposure during training and preventing robust detection.

**Comment 5:**

In Table 1, please add Celsius equivalents for body temperature (36.1–37.2 °C).

**Reply to 3rd Reviewer’s comment 5:**

According to the reviewer suggestions, In Table 1, I have added Celsius equivalents for body temperature and updated in revised manuscript as blue text.

**Comment 6:**

Correct minor language issues (e.g., “heterogeneity” vs. “heterogeneous”) and ensure consistent figure axis labels.

**Reply to 3rd Reviewer’s comment 6:**

The authors have taken this comment on borad and tried their best to address the minor language issues. And updated the heterogeneity to heterogeneous and ensure consistent figure axise labels like fig 11. I edit the axise labels.
